# Noncommunicable diseases and health system responses in Saudi Arabia: focus on policies and strategies. A qualitative study

**DOI:** 10.1186/s12961-022-00872-9

**Published:** 2022-06-13

**Authors:** Ahmed Hazazi, Andrew Wilson

**Affiliations:** 1 Menzies Centre for Health Policy and Economics, Sydney School of Public Health, The University of Sydney, No. 2W21/Level 2, Charles Perkins Centre D17, Sydney, NSW 2006 Australia; 2 Department of Public Health, Faculty of Health Sciences, Saudi Electronic University, Riyadh, Saudi Arabia

**Keywords:** Health policy, Health system, Noncommunicable diseases, Saudi Arabia

## Abstract

**Background:**

Noncommunicable diseases (NCDs) are responsible for an increasing disease and economic burden in Saudi Arabia, particularly those due to chronic diseases such as diabetes and cardiovascular disease. Efforts are being made to improve chronic disease control through greater prevention and disease management. This research examines the scope, comprehensiveness and perceived effectiveness of Saudi Arabia’s national policies and strategies to prevent and control NCDs and their risk factors.

**Methods:**

Semi-structured interviews were conducted with 25 managers of the Ministry of Health in Saudi Arabia. The interviewees were public health leaders, national programme directors and programme implementation staff. The interviews were transcribed and coded into key themes.

**Results:**

Interviewee responses indicated a belief that Ministry of Health programmes for the prevention and control of NCDs have achieved initial success, but have not yet been adequately evaluated. Interviewees reported faster development and implementation of policies for tobacco, sugar-sweetened drinks and obesity than for physical activity. Major challenges identified included inefficient programme management and low community awareness. There was a reported need for greater emphasis on health promotion and improving the effectiveness of existing multisectoral coordination.

**Conclusion:**

Effective national NCD policies and strategies have a critical role to play in the control of chronic disease epidemics. In Saudi Arabia, opportunities exist to improve the policy and strategies in response to NCDs by establishing a comprehensive surveillance system and linking epidemiological surveillance with health programme evaluation, as well as using a multisectoral and integrated approach. For better management and control of NCDs, a cohesive multisectoral collaboration with a comprehensive surveillance programme and adequate evaluation is urgently needed.

## Background

Noncommunicable diseases (NCDs) are among the main challenges facing healthcare systems globally, with additional social burden due to the major economic costs through loss of productivity. Globally, diabetes, respiratory disease, cardiovascular diseases and cancer account for over 80% of all premature deaths due to NCDs [1]. In Saudi Arabia, NCDs are responsible for 73% of all deaths [2]. Cardiovascular diseases are the leading cause of NCD deaths, accounting for 37% of total deaths, followed by cancer at 10%, then diabetes at 3%, respiratory diseases at 3% and other NCDs at 20% [3]. The impact of this global epidemic can be reduced through appropriate control measures. Preventing premature mortality, morbidity and disability requires reductions in chronic disease risk factors such as tobacco use, poor nutrition, physical inactivity and consumption of alcohol. To this end, the Saudi Arabian government, led by the Ministry of Health (MOH), has initiated new policies and programmes to improve prevention and treatment of NCDs.

Over the past two decades, the MOH has introduced four national initiatives to control the NCD epidemic. The first, in 2001, constituted the initial phase of tackling NCDs. A specialized committee was assigned to undertake the process of developing and updating the programme through several resolutions [4]. The second and third initiatives to tackle the increased prevalence of NCDs were introduced as Country Cooperation Strategies (2006–2011) and (2012–2016), which the Saudi Arabian MOH developed in cooperation with WHO [5, 6]. These strategies recommended that the Saudi Arabian healthcare system prioritize the concept of health promotion within the healthy lifestyle and prevention and control of NCDs. Also, as part of the recommendations, the strategies called for the development of an integrated programme for health education and research, which should be commensurate with the situation in Saudi Arabia.

The fourth initiative is the National Executive Plan for NCDs (2014–2025), which includes a comprehensive national plan to combat NCDs. This strategy aims to control and prevent NCDs from becoming more complicated [7]. This plan aligns with Gulf Cooperation Council (GCC) decisions on NCDs [8] and the WHO Global NCD Action Plan 2013–2020 to achieve the targets set by WHO for reducing premature mortality from NCDs among adults aged 30 to 70 years by 25% over a period of 15 years [9]. The global action plan provides guidance for Member States to initiate national NCD prevention strategies and support multisectoral actions in implementing preventive interventions called "best buy" interventions. The best buy interventions exist to reduce the burden of NCDs at the population level by targeting the shared risk factors. They include promoting physical activity, raising tobacco and alcohol taxes, enforcing tobacco and alcohol advertising bans, eliminating trans fat from food supply chains, reducing salt consumption, and detecting and treating NCDs at an early stage. WHO considered these interventions evidence-based, cost-effective and appropriate to implement within the health systems [9].

The Saudi Arabian government has implemented a wide range of policy measures in compliance with WHO objectives to strengthen the healthcare system, promote population health, and improve the management of NCDs [10]. The growing prevalence and impact of NCDs continue to challenge every health system, and effective national NCD policies and strategies have been demonstrated to reduce the NCD burden [9]. Although the national plan's objectives were relatively consistent with those of the 2013 WHO global action plans for preventing and managing NCDs, significant gaps were identified, including collaboration with the other governments, and the private sector was not mentioned. The plan lacked detailed information regarding policies, strategies and expected outcomes for preventing and monitoring NCD programmes. Additionally, the review of the literature indicated gaps in the NCD policies in the Saudi Arabian government plan. It also revealed that there has been limited scholarly consideration of the healthcare system's response to NCD management. Furthermore, there was a weakness in early detection, NCD prevention programmes (e.g. physical activity, healthy nutrition) and follow-up and coordination of care at primary healthcare centres [11–13]. This study aims to examine the response of the Saudi Arabian healthcare system to the management and control of NCDs and to identify the gaps from the perspective of the senior managers in the Saudi Arabian MOH.

## Objectives

As part of a mixed-methods study of the implementation of the Saudi Arabian national NCD plan, this paper reports on the interviews with senior managers in the Saudi Arabian MOH on the scope, comprehensiveness and perceived effectiveness of the national policies and strategies to prevent and control NCDs and their risk factors.

## Methods

### Study design

An exploratory qualitative study using semi-structured interviews was employed to understand the phenomena under investigation as experienced by the actors involved. This exploratory design allowed for the systematic collection, ordering, description and interpretation [14] of narratives generated from each interview. The philosophical worldview of pragmatism, whereby researchers focus on the defined research problem and how best to understand this using practical, outcome-oriented processes and embracing multiple viewpoints, was used [15]. After reviewing relevant literature on NCDs and the healthcare system in Saudi Arabia [16–19], a semi-structured interview guide was developed to explore the interviewees' views about the national policies and strategies developed by the MOH to prevent and control NCDs and their risk factors. The interview type was semi-structured to allow the interview to be responsive to respondents' answers and concepts emerging throughout the data collection process.

### Study setting and participants

This study was conducted in Riyadh, the capital city located in the central region of Saudi Arabia. Riyadh was chosen as the research context because it is the largest city with a population of more than 7 million and the business centre of Saudi Arabia, where most of the ministries and healthcare facilities are situated. A total of 31 key informants were identified through mailing lists and contacts from the MOH and were invited to participate in the interviews, of whom 25 agreed (response rate of 80%). The remaining invitees could not be contacted or did not reply to recruitment emails. A snowballing technique was employed where initial respondents identified other relevant colleagues who were referred to the study. Respondents were eligible to participate if they had executive health manager positions or worked experience in NCD management. All interviewees were contacted through email, with study information provided.

Interviewees were selected to contribute based on their expertise by using purposive and snowballing sampling to identify relevant informants for different programmes and management levels [20]. A sample of 25 managers from the Saudi Arabian MOH were interviewed. The interviewees reflected public health leaders, national programme directors and programme implementation staff from the MOH. Recruitment was ceased after 25 interviews as it was evident that no new themes were emerging (saturation) [21]. The interviews took place at the workplace office of each of the interviewees (as per their preference). All interviewees interviewed were willing to participate and responded to the interview questions. There was no refusal of consent or drop-out during interviews. We ensured that there was no social desirability bias in this study as there was no prior relationship between the interviewees and the interviewer.

### Data collection

Qualitative semi-structured interviews were undertaken to gather information about policies and strategies in relation to NCD prevention and management with stakeholders to attain the research aim. Open-ended interview questions were adopted to encourage a depth of understanding from each interviewee and to allow points of interest to be followed as they arose [14]. The interviewees' interview guide included questions regarding their views on MOH policies and strategies for the prevention and control of NCDs. Interviews were conducted by AH between May 2019 and August 2019. All interviews (*n* = 25) were conducted face to face at the workplace office of each of the interviewees, and the length of the interviews ranged from 30 to 55 min, with no repeat interviews conducted. Respondents' validation was achieved by returning transcripts to interviewees after completion of the interviews for comments or corrections. The approval to conduct this study was granted by the ethics committee of the MOH, Saudi Arabia (IRB log no: 2019-0028 E dated 02/04/2019).

### Data analysis

The interviews were audio-recorded, transcribed and coded into key themes. Informants were asked to recommend or provide any relevant policy statements or documents. A thematic analysis framework was used for analysing the data. This method consists of six steps: becoming familiar with the data, searching for themes, reviewing themes, defining and naming the themes, and finally, writing the research report [22]. The interview transcripts were reread to ensure that the identified themes formed a coherent pattern and captured the essence of the data. Transcripts were checked against audio files and entered into NVivo for management. NVivo software was used for organizing the data and facilitating the coding process. The first author (AH) generated the initial data analysis and set of codes, and the interpretation was modified and refined in discussion with the second author (AW). A final agreement on the key findings and results was reached in consultation by both authors. At the end of the coding process, a thematic map was created that identified the characteristics of the different themes, how they related to each other and how they represented the meanings evident in the data.

### Reporting the data

The design and reporting of the study were guided by the Consolidated Criteria for Reporting Qualitative Research (COREQ) checklist [23]. The analysis is presented with illustrative participant quotes to increase the transparency and reliability of the data presentation [23].

## Results

Themes and sub-themes are grouped according to whether they refer to national health policies, national programmes or future plans to prevent and control NCDs. Within national health policies, results are grouped by the major policy areas of nutrition, tobacco control and physical inactivity.

### National health policies

#### Nutrition policy

A range of policies led by the MOH and Saudi Food and Drug Authority (SFDA) were identified that are relevant to the healthy food strategy to eliminate nutrition-related risk factors for NCDs. These polices are as follows:

- limiting trans fatty acids (TFA) to 2% of the composition for fats and oils and 5% for other products, mandatory TFA labelling in the food supply which came into effect in 2015, and a ban on partially hydrogenated oils (PHOs) enacted in 2018;
- a 50% excise tax on sugar-sweetened drinks and 100% excise tax on energy drinks;
- mandatory nutrition calorie labelling of all menus in restaurants, cafes, fast-food chains and all other food stores which came into effect in 2019;
- a sodium limit of 1/100 g for bakery products;
- campaigns to encourage healthy food consumption to promote public health.

Interviewees noted that these reflect the objective of the Saudi Arabian government to comply and align legislation with the WHO global action plan for prevention and control of NCDs.

#### Physical activity policies

A strategic objective of the government’s Vision 2030 is to increase public participation in physical activities. This objective aims to improve health by reducing physical inactivity and other unhealthy lifestyle behaviours that are considered major risk factors for NCDs. Interviewees supported the importance of promoting physical activity as a key issue for the prevention of NCDs. Indeed, the majority pointed to sedentary living and physical inactivity as a critical issue for NCD prevention in Saudi Arabia. A common response was that while there are some existing initiatives promoting physical activity, there is a need for sustained health promotion programmes for increasing physical activity. The *Rashaqa* programme, a collaborative initiative between the Ministry of Education and the MOH focusing on increasing physical activity and preventing obesity by raising awareness among schools, was identified as an exemplar of what was required. Interviewees noted the need for a greater focus on policies and promotion of physical activity among adults, including greater attention to ensuring environmental changes that facilitated more physical activity.

#### Tobacco policies

Interviewees reported that in 2005, Saudi Arabia was one of the first countries to align its legislation with the WHO Framework Convention on Tobacco Control and subsequently formed a national committee for tobacco control in 2007. In 2017, under the Anti-Smoking Law issued by Royal Decree No. 56, the committee ratified a 100% tax on all tobacco products, banned smoking in public places, parks and workplaces, and banned tobacco advertisement and sponsorship. In parallel, the MOH increased other tobacco control measures such as public awareness campaigns, established clinical guidelines for smoking cessation, and increased access to the specialized clinics providing tobacco cessation therapy free of charge. In close sequence, in 2018, Saudi Arabia became the first country in the Middle East to enact plain packaging of tobacco products, effective from 2019. Also, in 2019, through the Ministry of Municipal and Rural Affairs and Housing, the government introduced a 100% surcharge tax added to the entire customer bill for coffee shops and restaurants that serve tobacco products.

Interviewees reported a notable incident following the introduction of plain packaging of tobacco, where tobacco consumers complained about a change in the flavour and quality of their same tobacco products, for which they blamed the SFDA regulations. Subsequently, the SFDA confirmed a difference in some flavour-related features in some tobacco products through independent laboratory testing. The SFDA asked the tobacco companies to modify these features to match the consumer's usual flavour.

### National programmes

#### Establishment of NCD control programmes

A range of programmes (Table 1) were developed to specifically reduce the incidence of NCDs and to improve the quality of life of populations. These programmes are applied in all primary healthcare centres and focus on cardiovascular diseases, diabetes, cancer, respiratory disease, obesity and cancer, mainly breast cancer and colon cancer. Interviewees reported that MOH programmes for the prevention of NCDs have led to an increase in counselling on healthy diet and on smoking cessation, and increased access to primary healthcare centres. The identified needs were effective programmes to promote healthy nutrition and physical activity and better access to dietitians/nutritionists.

**Table 1:** Summary of national programmes

Programme	Year launched	Description	Programmes strategies (applies to all programmes)
National programme for diabetes and prevention control	2013	The programme was designed to enhance the health awareness of the Saudi Arabian society regarding diabetes and its risk factors and to support the early detection and integrated healthcare programmes at all levels	Primary prevention Secondary prevention Quality health services Monitoring, evaluation and referral Research Societal partnership
National programme for obesity	2016	A 10-year programme aiming to stop the increasing rate of overweight and obesity according to the WHO global plan to combat chronic diseases 2013–2020	
National programme for cardiovascular diseases	2013	The programme was designed to enhance the health awareness of the Saudi Arabian society regarding cardiovascular disease and its risk factors	
National programme for cancer prevention	2012	The main goal of the programme is to reduce the mortality and morbidity of cancer and strengthen early detection	
National programme for asthma control	2012	The national programme for asthma prevention and control was designed to improve the level of care for asthma patients. This programme started in 2012 to be implemented in the primary healthcare centres; before this time it was in the hospitals only	

However, interviewees noted some issues with the implementation of these programmes. For example, one stated, *“We want to make sure to have the accurate statistics about the patients visiting the primary centres with chronic diseases as we noticed that some cases were not documented. Some centres documented the first visit for the patients only. But we will make sure that all cases are recorded…. Because in the programmes we need to know exactly how many patients are visiting the primary healthcare centres and benefiting from the programmes.”* Another interviewee stated, “*The studies done by the ministry found that majority of people do not visit the primary health centre, only if they are seeking treatment, the concept of regular check-up is absent which help us in early detection, so increasing outreaching programmes is important.”*

Interviewees stressed the importance of increasing outreach programmes and widening participation in the communities, and this could be achieved by increasing staff. Major challenges identified included inefficient programme management, low community awareness and the absence of structured screening programmes.

#### Multisectoral collaboration

Interviewees reported a lack of coordination between the health sector and other stakeholders. Therefore, they stressed the importance of multisectoral collaboration in improving NCD prevention, as the majority of NCD interventions occur outside the health sector. Most interviewees perceived a need to strengthen the national multisectoral policies by adopting a national master plan for multisectoral NCD prevention. It was reported that various sectors should implement the plan and clearly outline how related sectors coordinate with one another, and the plan should be effectively evaluated and monitored.

#### Monitoring and evaluation

Interviewees reported that the multiple policies implemented to reduce the incidence of NCDs were not being adequately evaluated. Most expressed a view that there is an ongoing need to periodically examine and evaluate the impact of the polices. It was reported that the NCDs programmes are evaluated internally within the MOH, and currently MOH is updating the guidelines for some programmes. Most interviewees reported that establishing a comprehensive database will increase the availability of accurate data and thereby facilitate programme evaluation, and that robust evaluation is needed to determine the effects of multiple policies for prevention programmes with regard to NCD outcomes in the short and long term.

### Future plans to prevent and control NCDs

#### Vision 2030

Interviewees reported that they believed there was political will within the Saudi Arabian government as reflected in the Saudi Vision 2030 for strengthening the healthcare system, improving healthcare facilities and providing quality healthcare services. The Saudi Vision 2030 is an ambitious reform plan formally approved and announced in 2016 [24]. Healthcare is one of the main focus areas of the Saudi Vision 2030 that seeks to improve the quality of healthcare services and facilities across the country [25]. As part of the broad strategy of Vision 2030, the MOH has medium-term and long-term strategies which include establishing a new model of care for NCD patients, and this model is intended to fill the gaps in health services that exist currently.

The gap was described by one interviewee as follows: “*Most of the chronic diseases patients in Saudi have insufficient knowledge about their diseases. The health services provided by the Ministry in the current form are fragmented and often is provided when necessary when the patient has complications*.” In relation to development of the new model of care another interviewee stated, *“We were asked to come in a new way to provide health services in a way that focuses on prevention in order to make the majority of society healthy… examples of these are health in all policy and healthy lifestyle in all aspects of life.”* Interviewees’ feedback highlighted the Saudi Vision 2030 emphasis on a healthy lifestyle and overcoming the risk factors for chronic diseases in order to improve prevention and treatment.

#### Chronic care model

The MOH is developing a new model of care for NCD patients which focuses on preventive care and early intervention to prevent diseases before they occur by empowering individuals and providing them with appropriate tools and information to support self-care using modern means of technology for communication. The model was described by an interviewee: “*In the new model, we are focusing on early detection, and patients have access to the specialist in a timely manner… we want to make sure that every patient has early diagnosis with a clear treatment plan.”* As part of the model, another interviewee reported that the MOH intended to maximize outreach programmes and empower people in schools, the workplace and communities.

## Discussion

This study was conducted to assess the scope, comprehensiveness and perceived effectiveness of the NCD prevention policy in Saudi Arabia from the perspective of senior health managers. Saudi Arabia has developed NCD strategic plans and policies which it believes are consistent with the WHO Global NCD Action Plan [9]. This study has shown that Saudi Arabia has implemented several important public health policies on NCD prevention. Interviewees perceived that efforts had been made to develop NCD prevention policies with a multisectoral approach used in policy development and implementation involving collaboration between the MOH, SFDA and other sectors. The study suggests that at least in the view of senior health managers, there is inconsistent progress in implementing all policies, with strong progress on nutrition and tobacco policies but less progress in public policy on promotion of physical activity. There are concerns that these policies and programmes have not been adequately evaluated, with the majority of the interviewees pointing out that the evaluation needs to be more robust and ongoing (or at least conducted periodically). Interviewees from different healthcare sectors also suggested the need for increased health awareness and that more campaigns may help this. Important gaps identified were lack of comprehensive surveillance programmes and structured screening programmes.

As indicated above, Saudi Arabia has a national strategy and has implemented polices on NCD prevention. In addition, it has developed and is implementing healthcare programmes to improve early detection and treatment of diseases and their risk factors through primary healthcare centres. This research shows the need for effective programmes to promote healthy nutrition and physical activity and better access to dietitians/nutritionists. Opportunities exist for multisectoral collaboration with nutritionists and dietitians in the private sector or other MOH facilities. Adopting nutrition referral schemes would be of value, for instance, formally referring patients to an accredited dietitian. Physicians acknowledge that providing nutrition counselling is part of their role [26]; however, they are not always able to provide sufficiently sustained and detailed nutrition advice that results in meaningful changes for their patients [27]. Collaboration between medical professionals and nutritionists is essential in addressing NCD risk factors [28]. Studies have shown the effectiveness and positive cost–benefit relationship of dietitian intervention, which reduces risk factors for NCDs and leads to improved blood pressure, glucose, lipids and weight, particularly when the dietitian is part of a multidisciplinary health team [29–32].

While there may be issues with the programme and policy evaluation, the policies are underpinned by a strategic and multisectoral approach. Policies that support increasing physical activity, improving healthy nutrition, and reducing tobacco consumption and harmful use of alcohol play a pivotal role in the prevention and control of NCDs and require multisectoral collaboration [9, 33, 34]. Multisectoral policies have been successful in a number of global settings when effectively applied to settings of everyday life [35–37]. Government fiscal policies such as taxation intended to decrease tobacco use or reduce sugar-sweetened beverage consumption have been successful in developed and developing countries [38, 39]. It is worth noting that Saudi Arabia appears to have introduced a range of such fiscal measures in tobacco control and improved nutrition. National health strategies that are embedded within NCD policy can ensure policy coherence as well as enabling coordination and collaborative actions of multi-stakeholder efforts outside the health sector [37].

One of the main achievements has been the implementation of comprehensive tobacco policy and controls under the leadership of the MOH and SFDA. Strong tobacco control policies have resulted in a significant decline in the prevalence of smoking over the past 10 years in developed countries [40]. Studies have reported that the alcohol, sugar-sweetened drinks and tobacco industries have often interfered with public health policy [41]. The incident reported here with tobacco product formulation appears to be another example of an attempt to undermine a government regulatory initiative in tobacco control.

In relation to nutrition policies, research has shown strong evidence linking NCDs to excessive salt consumption, high consumption of saturated fat, intake of trans fat and high consumption of sugary drinks [9]. In a number of global settings, multi-stakeholder efforts and government leadership on food labelling and reduction of salt and trans fat intake on a population-wide scale have been shown to be cost-effective [42]. Physical inactivity and sedentary behaviours also contribute to NCDs, including cardiovascular diseases, certain forms of cancer, type 2 diabetes mellitus and obesity [9]. Recent statistics indicated that 58.5% of the adult population in Saudi Arabia were physically inactive (67.7% of women and 52.1% of men) [43]. Ensuring an enabling environment that supports the promotion of physical activity and reduction of sedentary lifestyle among the Saudi Arabian population is critical to reducing the risks of NCDs. A mix of policies are in place in most developed countries [34]. Creating spaces that improve accessibility for walking and physical activity for all ages is crucial. This study shows that Saudi Arabian senior health managers believe there is a great need for additional policy, programme and promotional activity to address physical inactivity.

Opportunities exist to improve the policy and strategy responses to NCDs by linking epidemiological surveillance with health programme evaluation. Formal policy evaluation is important to understand the strengths and gaps in current policies, strategies and programmes, to determine what needs to be increased, added or eliminated. Monitoring of the health impact of the policy and programme for prevention and control of NCDs is essential in providing evidence for programme planning, policy development and evaluation. Interviewees in this study repeatedly identified the need to strengthen monitoring and evaluation, which will require improving health information systems and comprehensive and timely surveillance.

The limitation of the current evaluation is that the implemented NCD programmes and policies are evaluated internally within the MOH, and the implementation teams conduct the evaluation. It is possible that NCD programmes and policies have not yet been extensively evaluated, since the implementation teams may have a positive perception of the programmes and believe that the objectives set forth in the programmes have been met, or at the very least have been met to a great extent. Internal evaluators might find their objectivity compromised by the organization's policies and its underlying value system. Therefore, external evaluators are frequently perceived as being more objective than internal evaluators [44]. External evaluation specialists can provide more objective assessments of policies and programmes.

The MOH's capacity for policy development, implementation and evaluation is critical for the prevention and control of NCDs. A comprehensive database is required for the evaluation of NCD programmes and improved monitoring and management of NCDs. A comprehensive database contains administrative data, demographic data, patient medical history, health risks and health status, current management of health conditions, and outcomes data [45]. It is necessary to develop and implement a mechanism for collecting the relevant data from all healthcare institutions. Mechanisms should be developed to enable linkage between healthcare data systems, including the surveillance unit and electronic health records. Efforts to ensure that data on NCD risk factors, mortality and morbidity are used for policy formulation are essential for tracking the NCD burden and evaluating the effectiveness of programmes. These efforts should go beyond a one-time survey, to institutionalizing the surveillance and monitoring of NCDs within the health information system [9].

It would appear that considerable work is underway to reorient Saudi Arabian health services towards NCD prevention and control. Actions have been taken to develop a chronic care model with the aim of improving prevention, management and quality of services for NCDs. This is a crucial next step—a comprehensive model of care that focuses on promoting health and preventing NCDs and their risk factors and comprehensive management of NCDs across all health system levels. Ideally, this model(s) will be used to inform the investment in human resources and infrastructure that will be needed to deliver that model, including that required for equitable access to early detection, early treatment and interventions for preventing the recurrence of diseases [46].

## Recommendations

This study indicates a number of areas to improve the treatment and management of patients with NCDs. Improving the current evaluation is critical to assess the effectiveness and reach of policies and programmes and track any change in the burden of NCDs. The evaluation should identify two types of performance measures to be robust and determine the progress of NCD policies and programmes: process indicators, to show whether the processes have been implemented, and outcome indicators to show whether the objectives have been achieved. Robust evaluation improves both primary healthcare data and outcomes at the patient, health system and population health levels. It is crucial to regularly disseminate the findings related to the evaluation of the NCD policies and programmes to various stakeholders through publications to assist with ongoing programme improvement, report progress to stakeholders and to identify successful strategies.

Also, establishing a comprehensive surveillance system and linking epidemiological surveillance with health programme evaluation is likely to result in improved health outcomes and better value for the Saudi Arabian health system. A surveillance system provides the information needed to analyse how NCDs impact various populations by age and location, and tracks the progress of preventive efforts. Indeed, a comprehensive surveillance system is needed to integrate and extend current information across the multiple levels of decision-making to create actionable and timely knowledge for various stakeholders at the national level. Monitoring and surveillance of risk and protective factors for NCDs, and their prevalence for different population groups over time, are essential to inform the development, implementation and evaluation of the success of prevention interventions.

Multisectoral collaboration will be essential to activating partnerships with government and nongovernmental institutions, the private sector and civil society to promote health and control NCDs by establishing joint programmes and activities between the relevant authorities. Therefore, it emphasizes the significance of understanding partnership impacts, developing a shared vision, implementing a shared measurement system and creating opportunities for knowledge exchange that enable researchers, policy-makers and individuals working across multiple sectors outside of health to achieve multisectoral action for better health outcomes. Multisectoral collaboration aids in addressing NCD risk factors in a focused way by facilitating the pooling of resources and the formulation of shared objectives. One of the key benefits is the optimization of resource use by eliminating duplication of inputs and activities, which significantly increases the efficacy and efficiency of the NCD prevention programmes.

## Conclusion

The growing prevalence and impact of NCDs continues to challenge health systems worldwide, and effective national NCD policies and strategies have been demonstrated to reduce this burden of chronic diseases. Saudi Arabia has made efforts in policy development and implementation for NCD prevention and adoption of the WHO “best buy” interventions. This study suggests that the Saudi Arabian MOH and SFDA have developed and implemented major policies, strategies and programmes consistent with the WHO evidence-based best buys toward achieving the Saudi Vision 2030 and reducing NCDs. However, there is a need for more robust evaluation to assess the effectiveness and reach of policies and programmes and to monitor any change in the burden of NCDs. Based on this research, there is a reported need for greater emphasis on preventive measures and health promotion to minimize exposure to NCDs risk factors. Opportunities exist to improve the policy and strategic response to NCDs by establishing comprehensive surveillance systems and linking epidemiological surveillance with health programme evaluation, as well as using a multisectoral and integrated approach. Further research is suggested around more effective strategies to reduce physical inactivity and to address implementation gaps in other strategies.

## Data Availability

The datasets generated and/or analysed during the current study are not publicly available, as they contain interviews that were analysed into themes, but are available from the corresponding author on reasonable request.
